# Micronutrient deficiencies and the double burden of malnutrition in Vietnamese female adolescents: a national cross-sectional study in 2020

**DOI:** 10.1016/j.lanwpc.2024.101164

**Published:** 2024-08-07

**Authors:** Xiaomian Tan, Pui Yee Tan, Somphos Vicheth Som, Son Duy Nguyen, Do Thanh Tran, Nga Thuy Tran, Van Khanh Tran, J. Bernadette Moore, Yun Yun Gong

**Affiliations:** a School of Food Science and Nutrition, University of Leeds, Leeds, UK; b Section of International Health, Department of Health Sciences, Vrije Universiteit Amsterdam, Amsterdam, the Netherlands; c Division of Human Nutrition and Health, Wageningen University and Research, Wageningen, the Netherlands; d Nutrition Surveillance and Policy Department, National Institution of Nutrition, 48B Tang Ba Ho, Hai Ba Trung District, Ha Noi, Vietnam; e Department of Micronutrient, National Institution of Nutrition, 48B Tang Ba Ho, Hai Ba Trung District, Ha Noi, Vietnam

**Keywords:** Micronutrient deficiency, Double burden of malnutrition, Adolescents, Iron, Zinc, Vitamin A

## Abstract

**Background:**

Vietnam is facing a double burden of malnutrition, with increasing prevalence of overweight coexisting with undernutrition (stunting and/or thinness) and micronutrient deficiencies (MNDs). Although malnutrition during female adolescence leads to poor health outcomes with potential intergenerational effects on offspring, no studies have comprehensively investigated MNDs and nutritional status among contemporary Vietnamese female adolescents.

**Methods:**

Data from 10- to 18-year-old female participants (n = 1471) in the nationally-representative Vietnam General Nutrition Survey 2020 were analysed. Blood nutritional biomarkers, anthropometric measurements, and sociodemographic data were collected, and associations between nutrition status and MNDs were analysed; with anaemia, iron deficiency (ID), iron deficiency anaemia, low serum zinc, low serum retinol, and any MNDs as specified outcomes.

**Findings:**

Prevalence of overweight, stunting, and thinness was 27.2%, 14.3%, and 6.9%, respectively. Low serum zinc was common (39.8%), as was ID (13.4%). Bivariate analyses showed that older age (16–18 years old), ethnic minority status, lower wealth index, and inflammation were associated with MNDs. In adjusted logistic regressions, stunting was associated with increased odds ratio and [95% confidence intervals] of low serum retinol (8.92 [2.26, 35.15], p < 0.01), as was thinness (12.25 [3.47, 43.33], p < 0.01). Stunting was also associated with increased odds of having any MND (2.06 [1.31, 3.25], p < 0.01).

**Interpretation:**

More female adolescents were overweight than undernourished in Vietnam in 2020. However, undernutrition, low serum zinc, and ID remain prevalent. Food systems approaches should be considered to stem the stark increase in the double burden of malnutrition in young people living in Vietnam.

**Funding:**

UK BBSRCBB/T008989/1.

**Research in context**

**Evidence before this study**

Malnutrition during female adolescence leads to poor health outcomes and has the potential for intergenerational effects on offspring. We searched PubMed from inception to February 1, 2024 for studies that had investigated micronutrient deficiencies (MNDs) or nutritional status (both over and undernutrition) in female adolescents living in Vietnam using the search terms “Vietnam∗” and (“female∗” or “girl∗”) and “adolescent∗” and (“double burden of malnutrition” or “micronutrient∗” or “iron” or “zinc” or “vitamin A” or “overweight” or “obese” or “thinness” or “stunting” or “underweight”) with no language restriction. Of 188 studies identified, 104 had been published in last 10 years. These studies most frequently reported regional-specific data on body weight (City Ho Chi Minh or specific rural areas), as well as results from the Vietnamese cohorts in the Young Lives prospective cohort study, which included children born in either 1994/5 or 2001/2 that were followed longitudinally for over 15 years since 2001. Although a few studies reported on the prevalence of stunting and iron deficiency in comparison with other Southeast Asian countries, we found no studies that had comprehensively investigated both micronutrient and nutritional status in contemporary Vietnamese adolescents.

**Added value of this study**

This is the first study to report the prevalence of micronutrient deficiencies and the double burden of malnutrition in female adolescents living in Vietnam in 2020. Our study highlights the current double burden of malnutrition in Vietnam, with a higher prevalence of overweight (27.2%) than undernutrition (stunting and/or thinness, 17.3%) existing among contemporary female adolescents. In addition, our results show regional, residential, ethnic, and wealth index disparities in undernutrition and MNDs. Not least, our study suggests that iron deficiency and low serum zinc levels remain prevalent among Vietnamese female adolescents.

**Implications of all the available evidence**

Additional efforts on implementation of food fortification policies, food-based interventions, targeted supplementation, and nutrition education should be made for at risk female adolescents living in Vietnam. Food environment and food systems approaches should be considered to stem the recent stark increase in overweight in young people living in Vietnam and other Western Pacific nations, such as the Philippines and Cambodia, newly facing the nutrition transition and double burden of malnutrition.

## Introduction

While considerable progress has been made in reducing the global burden of micronutrient deficiencies (MNDs) in recent decades, the prevalence of MNDs remains high in some populations.^1^ Concerningly, an estimated 56% of children under five years of age, and 69% of women of reproductive age (WRA, 15–49 years) globally are affected by at least one of a few, very common, MNDs.^2^ In particular, iron, zinc, and vitamin A deficiencies are the most common MNDs worldwide, and are causally associated with adverse health outcomes and the global burden of disease.^1^^,^^2^ Following infants and toddlers, female adolescents represent one of the most susceptible developmental and population age groups to MNDs.^3^

Indeed, after infancy and early childhood, adolescence is increasingly recognised as a unique and critical period for human growth and development, with profound hormonal, physical, and psychosocial changes.^4^ Poor nutrition during adolescence impacts full height development, quality of later life; and, in the case of adolescent females, has intergenerational effects on offspring that perpetuate an ongoing cycle of malnutrition.^3^^,^^4^ Approximately 1.8 billion individuals worldwide are adolescents between 10 and 19 years old, with 90% of them living in low- and middle-income countries (LMICs).^4^ In general, females have higher risk of MNDs such as iron deficiency (ID) and iron deficiency anaemia (IDA). This is because in addition to differences in reproductive biology, females are more susceptible to discriminative cultural, social, and gender norms.^3^^,^^4^ These discriminatory norms manifest in increased poverty, illiteracy, inequality, and early marriage, which individually and collectively increase risk for MNDs in female adolescents globally, and often particularly in LMICs.

Similar to other LMICs, Vietnam is facing a double burden of malnutrition, that is, the co-existence of under- and overnutrition at community and country levels.^5^ Economic development and rapid changes to food environment have resulted in a nutrition transition with a marked increase in the prevalence of overweight and obesity in childhood.^5^^,^^6^ Among 5–19-year-olds in Vietnam, the prevalence of overweight and obesity increased from 8.5% (2010) to 19% (2020).^7^ In Ho Chi Minh city, although the prevalence of stunting and thinness has decreased in recent decades, nonetheless undernutrition remained high at 6–14% for high school students in 2014; while 19% were overweight or obese.^5^ Similarly, although multiple national nutrition strategies aimed at improving population micronutrient status have been implemented,^8^^,^^9^ with consequent reductions in some MNDs, nonetheless the prevalence of some MNDs remains stubbornly high. For example, the pooled prevalence of anaemia in Vietnamese WRA decreased from 42.6% (1995) to 16.9% (2013),^10^ but in 2010 zinc deficiency was noted to still be of severe public health significance, with more than 60% of WRA and children in Vietnam having low serum zinc.^11^

Given the rapid nutrition transition unfolding in Vietnam, the aims of this study were to examine: 1) the prevalence of different forms of malnutrition including undernutrition, overnutrition and MNDs (ID, low serum zinc and retinol); and 2) the associations between MNDs, demographic and socioeconomic factors, and growth indices; in female adolescents aged 10–18 years old, utilising the data from the nationally-representative, Vietnam General Nutrition Survey (GNS) 2020. Our hypothesis was that the risk of MNDs would be indicated by abnormal growth and associated with sociodemographic factors in female adolescents living in Vietnam.

## Methods

### Study design

To understand the associations between MNDs and growth indices, secondary data analyses were performed on GNS 2020 data following the Strengthening the Reporting of Observational studies in Epidemiology (STROBE) guidelines. Conducted by the National Institute of Nutrition of Vietnam every 10 years, the GNS is a nationally-representative, population-based, cross-sectional survey that combines anthropometric and blood biomarker measurements with comprehensive sociodemographic data. In order to prevent selection bias, participants were recruited using a multi-stage cluster sampling design. Clusters corresponding to census enumeration areas (EAs) were treated as primary sampling units. EAs were selected from 25 provinces to represent the six geographical regions in Vietnam, namely the: 1) Northern midlands and mountainous areas, 2) Central highlands, 3) Red River Delta (including Hanoi, the Capital); 4) North–central and central coastal areas, 5) Southeast, and the 6) Mekong River Delta (including Ho Chi Minh City). The EAs were allocated to either an urban or rural stratum to ensure representation of urbanicity. Lastly, eligible individuals from each EA were randomly selected for enrolment in GNS 2020, which included 143 communes (wards) based on probability proportional to size method.

Of the 20,864 participants completing the GNS 2020, a total of 1471 participants met our inclusion criteria of being aged 10- to 18-years-old and female, and were included in this study for analysis (Supplementary Fig. S1, participant flow diagram).

### Ethics statement

The GNS 2020 was reviewed and approved by the Ethical Committee of the National Institute of Nutrition, Vietnam. All methods were conducted in accordance with the guidelines laid down in the Declaration of Helsinki and written informed consent was obtained from each participant prior to data collection.

### Anthropometric measurements

Procedures for taking participant’s height and weight measurements in the GNS 2020 have been described in detail (preprint).^12^ Body mass index (BMI) was calculated as weight (kg) divided by square of height (m^2^). The World Health Organization (WHO) growth reference was used to define stunting, thinness, and overweight.^13^ Stunting was defined by height-for-age z-score (HAZ) < −2 SD, while thinness and overweight were defined by BMI-for-age z score (BAZ) < −2 SD; and BAZ > +1, respectively. Outliers with HAZ > +6 SD or < −6 SD, or BAZ > +5 SD or < −5 SD were excluded from the dataset.

### Blood biomarkers and definition of micronutrient deficiencies

Blood samples were taken as part of the Vietnam GNS 2020 as detailed (preprint),^12^ and aliquoted for storage prior to biomarker measurements, while haemoglobin was measured at point-of-care (Hb 301 System; HemoCue, Sweden). Serum: ferritin, transferrin receptor, retinol binding protein, C-reactive protein (CRP), and alpha-1-acid glycoprotein (AGP) were measured by sandwich enzyme-linked immunosorbent assay at the VitMin Lab (Germany). While serum retinol was measured by reverse-phase liquid chromatography with tandem mass spectrometry (3200 QTRAP, Sciex U.S.A), and serum zinc by atomic emission spectrometry (Avanta+; GBC Scientific Equipment, Australia). Diagnostic cut-offs followed the International Zinc Nutrition Consultative Group (IZiNCG)’s guidelines for low serum zinc, and the WHO micronutrient survey manual for ID, IDA, low serum retinol and inflammation.^14^^,^^15^ Anaemia was defined as haemoglobin concentration levels <115 g/L and <120 g/L for 10–11 years old and 12–18 years old, respectively. For ID, the cut-offs of serum ferritin concentration <15 μg/L and 70 μg/L were applied for apparently healthy individuals and individuals with inflammation (CRP > 5 mg/L and/or AGP > 1 g/L), respectively.^14^ The Biomarkers Reflecting Inflammation and Nutritional Determinants of Anaemia (BRINDA) correction method was performed for serum ferritin in the R environment (R-4.2.3) with established pipelines.^16^ IDA was defined as the co-existence of anaemia and ID. Low serum zinc was defined as serum zinc under different conditions: serum zinc <10.7 μmol/L (morning, fasting), <10.1 μmol/L (morning, non-fasting) or <9.0 μmol/L (afternoon, non-fasting). Low serum retinol was defined as serum retinol concentration <0.70 μmol/L. Any MNDs were defined as having at least one of any of the MNDs described above (ID, low serum zinc, and low serum retinol) and were reported as having 1 or ≥2 MNDs. To avoid bias in measurement, in addition to utilising the aforementioned standardised definitions and cut-offs from WHO and IZiNCG, within-assay and between-assay variability tests were performed for quality control of the biomarker measurements.

### Demographic and socioeconomic indicators

Demographic and socioeconomic data were collected using structured questionnaires. These included age, sex, geographical regions, urban or rural demographics, ethnicity (Kinh or a minority ethnic group, e.g., Tay, Thai, Muong, Khmer, Nung, Hmong or other), and household wealth index (quintiles). The details of the wealth index calculation have been reported (preprint).^12^

### Statistical analyses

Descriptive and logistic regression analyses were performed using complete case analysis, (excluding participants with missing data) in STATA 17 (Stata Corporation, College Station, TX, US). The number and percentage of missed observations for each variable are listed in Supplementary Table S1. All analyses were performed based on sampling weights, using the svy prefix. Descriptive analyses for the demographic, socioeconomic, anthropometric and micronutrient status were reported for total samples and by nutritional status (stunting, overweight and thinness). Quantitative data are presented as mean ± SD for continuous variables and number (%) for categorical variables, with the exception of serum ferritin levels, which are presented as geometric mean. Given the large sample size, the Wald and Chi–Square tests were performed on continuous and categorical variables respectively, to assess the difference of characteristics of participants among nutritional status, unless specifically noted.

Bivariate logistic regression was first performed to assess the associations between MNDs (as outcomes) and growth indices and sociodemographic indicators, with the intention of finding the significant associations and then further estimating the associations between MNDs and growth indices in multivariate regression, adjusting for those variables that showed significant association with MNDs, as well as variables previously reported to be risk factors of MNDs (e.g., area of residence) in the preprint.^12^ Ultimately, we adjusted for the potential covariates: age group (done categorically for groups 10–12, 13–15, 16–18 years old), inflammation and sociodemographic factors (area of residence, ethnicity and wealth index).

Crude (COR) and adjusted odds ratio (AOR) with 95% confidence intervals (CI) were reported for binominal outcomes (5 individual MNDs), while crude and adjusted relative risk ratio (RRR) with 95% CI were reported for multinominal outcomes (interpreted similarly to the odds ratio as comparison of exposure; i.e., having 1 or ≥2 MNDs, relative to reference of no MNDs). Significance level was set at 0.05. Sensitivity analyses were done by: 1) comparing subsamples (50% of the total population, randomly selected) within the total sample; and 2) testing 2 different logistic regression models with or without inflammation status as a covariate. Data visualisation was conducted using ggplot2 in the R environment (R-4.2.3).

### Role of the funding source

The funders had no involvement in study design, data analysis, manuscript writing, interpretation or decision to submit the paper for publication.

## Results

### Characteristics of participants

Data from 1471 female adolescent participants in the Vietnam GNS 2020, aged 10–18 years old were included in this study. The overall prevalence of overweight, stunting, and thinness was 27.2%, 14.3%, and 6.9%, respectively (Table 1). Sociodemographic factors investigated in relation to nutritional status included geographical areas, urban or rural areas, ethnicity, and wealth index. These were all associated with stunting (p < 0.01, Table 1). Stunting was more prevalent in: adolescents living in northern Vietnam (the northern mountains and north central & central coastal regions) and rural areas, ethnic minorities, and those living in households with a low wealth index. In contrast, a high prevalence of both overweight (24.4%) and thinness (22.4%) was found among females who lived in Delta and Hanoi. The highest prevalence of thinness (25.0%) was from participants living in Mekong delta, and the overall prevalence of undernutrition was 17.3% (either stunting, thinness, or both). Prevalence of overweight was low in Northern mountains (8.6%), while thinness prevalence (15.3%) was high in this area (Table 1). The average age of the study participants was 12.8 ± 2.2 years old, although adolescents with stunting were older than those without stunting (13.3 ± 2.6 versus 12.6 ± 2.1, p < 0.01). While thin adolescents were younger than those with normal weight (12.1 ± 2.0 versus 13.0 ± 2.2, p < 0.01).

**Table 1:** Socio-demographic characteristics of female adolescents living in Vietnam between 10 and 18 years old (n = 1471).

	Total (n = 1471)	Non-stunted (n = 1068; 85.7%)	Stunted (n = 185; 14.3%)	p value	NW (n = 967; 65.9%)	OW (n = 402; 27.2%)	Thinness (n = 102; 6.9%)	p value
Age in years, mean ± SD	12.8 ± 2.2	12.6 ± 2.1	**13.3 ± 2.6**	**<0.01**^a^	13.0 ± 2.2	12.6 ± 2.2	**12.1 ± 2.0**	**<0.01**^b^
10–12 y	721 (49.0%)	557 (87.6%)	74 (12.4%)	**<0.01**	462 (61.0%)	194 (29.7%)	65 (9.4%)	**<0.01**
13–15 y	548 (37.3%)	375 (86.9%)	64 (13.1%)		364 (69.4%)	153 (25.6%)	31 (5%)	
16–18 y	202 (13.7%)	116 (73.2%)	47 (26.8%)		141 (75.3%)	55 (22%)	6 (2.7%)	
**Geographical region, n (%)**								
Northern mountains	208 (14.5%)	132 (13.1%)	57 (30.4%)	**<0.01**	159 (16.9%)	38 (8.6%)	11 (15.3%)	0.51
Delta and Hanoi	306 (26.1%)	265 (28.5%)	19 (14.1%)		233 (27.1%)	56 (24.4%)	17 (22.4%)	
North Central & Central Coast	262 (19.1%)	188 (18.2%)	40 (26.6%)		174 (19.6%)	69 (17.6%)	19 (19.8%)	
Highlands	271 (8.0%)	151 (6%)	34 (9%)		140 (6.3%)	110 (11.9%)	21 (9.4%)	
Southeast and City. Ho Chi Minh	220 (16.2%)	168 (15.8%)	16 (10.4%)		138 (14.9%)	74 (21.4%)	8 (8.2%)	
Mekong Delta	204 (16.1%)	164 (18.4%)	19 (9.5%)		123 (15.1%)	55 (16.1%)	26 (25%)	
**Area of residence, n (%)**								
Urban	614 (35.7%)	454 (36.2%)	48 (19.5%)	**<0.01**	377 (32.3%)	199 (44.2%)	38 (34.1%)	0.10
Rural	857 (64.3%)	614 (63.8%)	137 (80.5%)		590 (67.7%)	203 (55.8%)	64 (65.9%)	
**Ethnicity, n (%)**								
Kinh	1171 (82.2%)	907 (87.7%)	96 (58.8%)	**<0.01**	768 (82.9%)	325 (80.6%)	78 (81.6%)	0.82
Minorities	300 (17.8%)	161 (12.3%)	89 (41.2%)		199 (17.1%)	77 (19.4%)	24 (18.4%)	
**Wealth index, n (%)**								
Lowest	247 (17.5%)	149 (15.5%)	67 (31.8%)	**<0.01**	177 (18.6%)	53 (14.6%)	17 (18%)	0.71
Second	304 (17.4%)	211 (17%)	48 (22.2%)		187 (17%)	87 (17.2%)	30 (22.1%)	
Third	323 (22.2%)	228 (21.7%)	34 (23.9%)		198 (21.3%)	100 (22.9%)	25 (27.9%)	
Fourth	351 (25.2%)	273 (26.3%)	22 (14.5%)		227 (24.6%)	109 (27.8%)	15 (20.2%)	
Highest	246 (17.8%)	207 (19.4%)	14 (7.7%)		178 (18.5%)	53 (17.5%)	15 (11.8%)	

Data are presented as mean ± SD for continuous variables and n (%) for categorical variables.

Wald test and Chi square test were performed for continuous variables (age in year) and categorical variables (age groups, geographical region, area of residence, ethnicity and wealth index), respectively.

NW: normal weight; OW: overweight.

a P value was calculated comparing with non-stunted group using Wald test.

b P value was calculated comparing thinness group with NW group by Wald test. The age between OW and NW group was not significantly different (p > 0.05).

Interestingly, no differences were observed in the levels of serum biomarkers between stunted and non-stunted adolescents (Table 2). However, both serum retinol and retinol binding protein levels were significantly lower in adolescents who were thin compared to those with normal weight (p < 0.01, Table 2). While 5.9% of participants exhibited markers of inflammation, these indicators did not vary significantly in relation to nutritional status. No differences were observed in blood biomarkers between the overweight and normal weight groups. Notably, serum ferritin levels were higher in participants with thinness (p < 0.01), but remarkably ID was not associated with body weight status (Table 2). Prevalence of low serum retinol was much higher in adolescents with thinness compared to those with overweight and normal weight (7.0% versus 1.7% and 0.8%; p < 0.01); and was found more prevalent among adolescents with stunting than those who were not stunted (3.8% versus 1.0%, p = 0.04). Moreover, having any individual or combination of MNDs were associated with stunting (p < 0.01) but not thinness or overweight (Table 2).

**Table 2:** Anthropometric, biochemical characteristics and micronutrient status of female adolescents living in Vietnam between 10 and 18 years old (n = 1471).

	Total (n = 1471)	Non-stunted (n = 1068; 85.7%)	Stunted (n = 185; 14.3%)	p value^a^	NW (n = 967; 65.9%)	OW (n = 402; 27.2%)	p value^b^	Thinness (n = 102; 6.9%)	p value^b^
**Anthropometric**									
HAZ	−0.94 ± 1.07	−0.65 ± 0.82	−2.67 ± 0.62	**<0.01**	−0.99 ± 1	−0.46 ± 1.2	**<0.01**	−1.38 ± 1.07	**0.02**
BAZ	−0.31 ± 1.22	−0.23 ± 1.2	−0.78 ± 1.21	**<0.01**	−0.42 ± 0.79	1.56 ± 0.46	**<0.01**	−2.61 ± 0.52	**<0.01**
**Biomarkers**									
Haemoglobin (g/dL)	13.1 ± 1.0	13.1 ± 1.0	12.8 ± 1.3	0.09	13.1 ± 1.0	13.1 ± 1.0	0.39	12.9 ± 1.0	0.13
Serum ferritin (μg/L) (geometric mean ± SE)^c^	45.8 ± 1.0	47.3 ± 1.3	44.6 ± 4.6	0.60	45.6 ± 1.7	43.7 ± 1.6	0.54	57.7 ± 4.3	**<0.01**
Serum transferrin receptor (mg/L)	5.9 ± 2.6	5.71 ± 1.85	6.6 ± 4.6	0.13	5.9 ± 2.6	5.9 ± 2.6	0.77	5.6 ± 2.3	0.26
Serum zinc (μmol/L)	11.4 ± 5.3	11.4 ± 5.2	11.3 ± 6.2	0.89	11.4 ± 5.5	11.5 ± 5.1	0.72	11.1 ± 4.4	0.74
Serum retinol (μmol/L)	1.3 ± 0.3	1.3 ± 0.29	1.2 ± 0.3	0.43	1.3 ± 0.3	1.8 ± 0.3	0.58	1.1 ± 0.3	**<0.01**
RBP (μmol/L)	1.8 ± 0.4	1.37 ± 0.3	1.4 ± 0.4	0.55	1.4 ± 0.5	1.4 ± 0.4	0.77	1.2 ± 0.3	**<0.01**
CRP (mg/L)	0.6 ± 2.8	0.6 ± 2.5	0.9 ± 5.0	0.56	0.5 ± 2.6	0.9 ± 2.7	0.12	0.9 ± 4.5	0.46
AGP (mg/L)	0.6 ± 0.3	0.6 ± 0.2	0.6 ± 0.3	0.34	0.6 ± 0.2	0.6 ± 0.3	0.13	0.5 ± 0.3	0.23

MNDs								
	Total (n = 1471)	Non-stunted (n = 1068; 85.7%)	Stunted (n = 185; 14.3%)	p value	NW (n = 967; 65.9%)	OW (n = 402; 27.2%)	Thinness (n = 102; 6.9%)	p value
Anaemia	143 (9.5%)	94 (8.8%)	29 (14.8%)	0.07	97 (10%)	34 (7.1%)	12 (13.8%)	0.14
ID	169 (13.4%)	128 (12.2%)	25 (14.4%)	0.57	124 (13.3%)	55 (15.2%)	7 (6.9%)	0.29
IDA	53 (3.8%)	39 (3.1%)	11 (5.5%)	0.12	40 (3.7%)	19 (4.4%)	2 (2.2%)	0.64
Low serum zinc	563 (39.8%)	397 (39.5%)	83 (49.6%)	0.11	375 (41.5%)	151 (35.1%)	37 (42.5%)	0.21
Low serum retinol	16 (1.5%)	9 (1.0%)	3 (3.8%)	**0.04**	7 (0.8%)	6 (1.7%)	3 (7.0%)	**<0.01**
Chronic or acute inflammation	82 (5.9%)	57 (5.3%)	12 (6.8%)	0.51	49 (4.6%)	28 (9%)	5 (5%)	0.09
**Number of any MNDs**^d^								
0	457 (52.8%)	359 (54.9%)	39 (35.9%)	**0.05**	298 (51.1%)	127 (57.7%)	32 (47.8%)	0.26
1	363 (41.5%)	266 (40.2%)	46 (53.2%)		242 (43.1%)	98 (36.6%)	23 (46.8%)	
2	43 (5.0%)	27 (4.3%)	9 (9.7%)		30 (5.5%)	11 (4.6%)	2 (2.3%)	
3	6 (0.7%)	3 (0.5%)	1 (1.3%)		2 (0.3%)	3 (1.0%)	1 (3.1%)	

Data are presented as mean ± SD for continuous variables and n (%) for categorical variables unless specifically noted.

Wald test and Chi square test were performed for continuous variables (anthropometric, biomarkers) and categorical variables (MNDs and number of any MNDs), respectively.

AGP: alpha-1 acid glycoprotein; BAZ: BMI-for-age z score; BRINDA: Biomarkers Reflecting Inflammation and Nutritional Determinants of Anaemia; CRP: C-reactive protein; HAZ: height-for-age z score; NW: normal weight; OW: overweight; RBP: retinol binding protein.

a P value was calculated comparing with non-stunted group.

b P value was calculated comparing with normal weight group.

c P value was calculated based on a univariate regression model; serum ferritin data was corrected with BRINDA method.

d Participants with numbers of any following MNDs: ID, Low serum zinc and retinol.

### Prevalence of malnutrition and micronutrient deficiencies

**Fig. 1:**
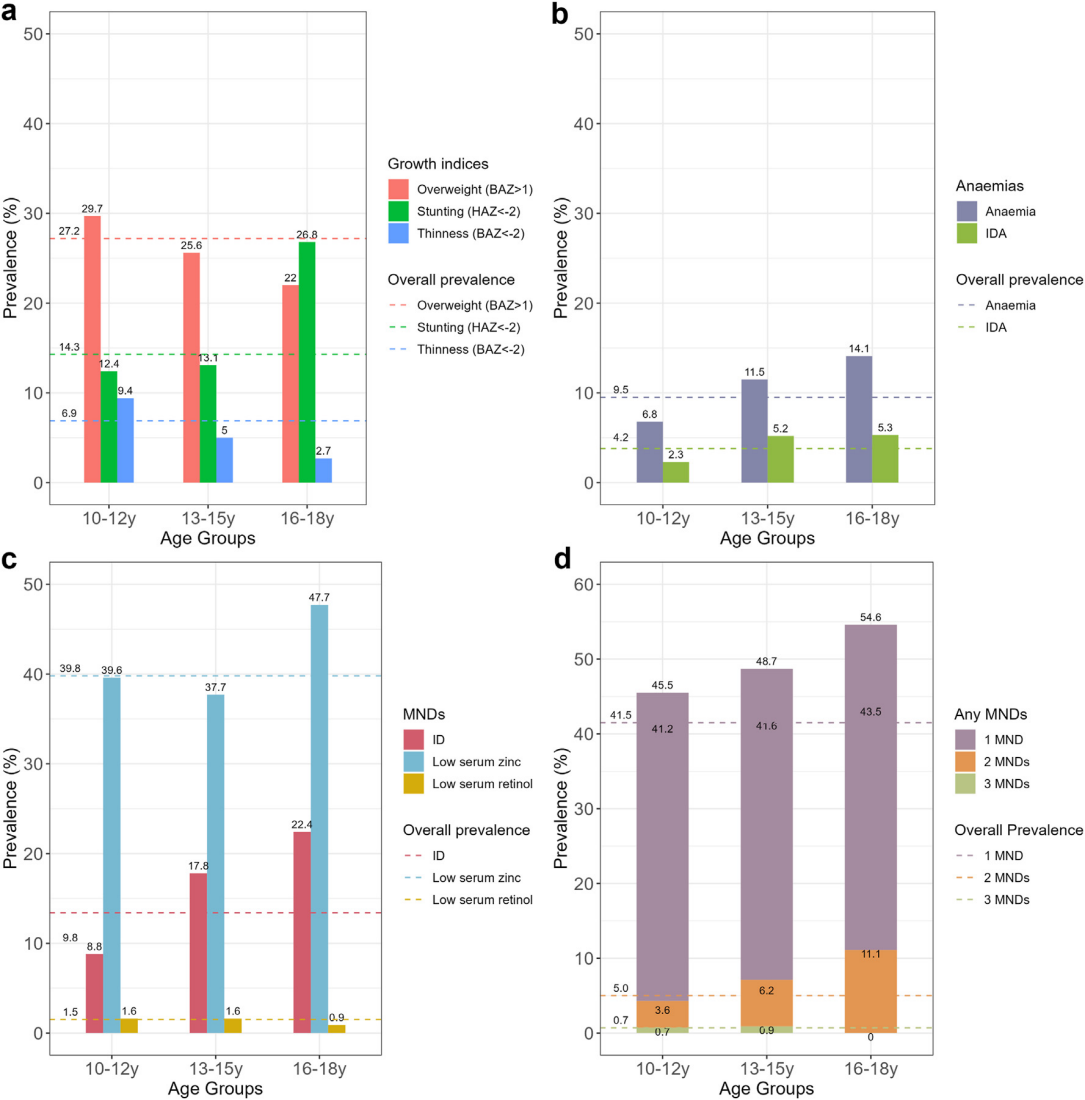
Prevalence of malnutrition in female adolescents aged 10–18 years old living in Vietnam in different age groups. Data show the prevalence of overweight, stunting and thinness **(a)**, anaemias **(b)**, MNDs **(c)** and distribution of any MNDs (any of ID, low serum zinc or low serum retinol) **(d)**. Prevalence was estimated based on sampling weight. Dash lines represented the overall prevalence. ID: iron deficiency; IDA: iron deficiency anaemia; MNDs: micronutrient deficiencies.

Notable differences in nutritional status and MNDs emerged when participants were stratified into early-adolescent (10–12 years old), middle-adolescent (13–15 years old) and late-adolescent (16–18 years old) age groups (Fig. 1). Prevalence of both overweight and thinness was higher in early-adolescents compared to late-adolescents (29.7% versus 22.0%, 9.4% versus 2.7%, respectively; Fig. 1A). Whereas, compared to early-adolescents, the prevalence of stunting was higher in the late-adolescent group (26.8% versus 12.4%; Fig. 1A). Anaemia prevalence was lowest in the early-adolescent group (6.8%), but more common in late-adolescents (14.1%; Fig. 1B). Similarly, the prevalence of IDA was dramatically higher in middle- (5.2%) and late- (5.3%) adolescents in comparison to early-adolescent females (2.3%; Fig. 1B). Both ID and low serum zinc were more prevalent in the late-adolescents, whereas low serum retinol was less prevalent in the oldest group (Fig. 1C). Low serum zinc was the most common MND, affecting 39.8% of the study participants, particularly in late-adolescents (47.7%; Fig. 1C). Only 1.5% of the female adolescents had low serum retinol levels (Fig. 1C). When we examined the existence of any MNDs, nearly half (40.8%) of all female adolescents in the GNS 2020 had at least one MND (Fig. 1D). The prevalence of having ≥1 MNDs was lowest in early-adolescents (45.5%) and highest in late-adolescents (54.6%; Fig. 1D). Notably, late-adolescents had a much higher prevalence of having ≥2 MNDs (11.1%) compared to early-adolescents (3.6%). Only a very small proportion (0.7%) of study participants had three MNDs (Fig. 1D).

Stark differences in the nutritional status and prevalence of MNDs existed between adolescents from rural areas in comparison to urban areas (Supplementary Figs. S2–S4). The prevalence of overweight was much higher in adolescents from urban areas (33.7 versus 23.6% in rural areas; Supplementary Fig. S2), while stunting prevalence was much higher in females from rural areas (17.4 versus 8.3%; Supplementary Fig. S2). The prevalence of ID was much higher in rural adolescents from rural areas compared to those from urban areas, particularly 16–18 year-olds (32% versus 8.2%; Supplementary Fig. S3), as was ≥2 MNDs in all age groups (Supplementary Fig. S4).

### Associations between micronutrient deficiencies, sociodemographic indicators and growth indices

Unadjusted binominal and multi-nominal logistic regressions were performed to examine the association between MNDs (as outcomes; individually and combined) and age, ethnicity, area of residence, wealth index and growth indices (Table 3). The associations between MNDs and growth indices were then further estimated while adjusting for other variables (age group, ethnicity, geographical regions) that showed significant association with MNDs in unadjusted analyses and variables reported to be risk factors of MNDs in previous studies (area of residence; Table 4).

**Table 3:** Unadjusted binominal and multi-nominal logistic regression between micronutrient status and body weight status among female adolescents aged 10–18 years old living in Vietnam (n = 1471).

	Individual MNDs^a^					Any MNDs^b^	
	Anaemia	ID	IDA	Low serum zinc	Low serum retinol	1 MND	≥2 MNDs
	COR [95% CI]	COR [95% CI]	COR [95% CI]	COR [95% CI]	COR [95% CI]	CRRR [95% CI]	CRRR [95% CI]
Age group (ref:10–12 y)	1	1	1	1	1	1	1
13–15 y	1.78 [0.94, 3.38]	**2.24 [1.44, 3.47]**^d^	2.30 [0.83, 6.36]	0.93 [0.73, 1.17]	1.00 [0.25, 4.04]	1.07 [0.77, 1.49]	1.77 [0.66, 4.74]
16–18 y	**2.24 [1.40, 3.60]**^d^	**2.99 [1.70, 5.27]**^d^	2.32 [0.85, 6.32]	1.39 [0.88, 2.20]	0.54 [0.04, 6.93]	12.7 [0.51, 3.19]	**3.15 [1.02, 9.80]**^c^
Ethnicity (ref: Kinh)	1	1	1	1	1	1	1
Minorities	1.81 [0.92, 3.54]	0.90 [0.42, 1.94]	1.64 [0.59, 4.53]	**1.63 [1.08, 2.47]**^c^	1.47 [0.56, 3.81]	1.39 [0.93, 2.09]	1.78, 0.70, 4.57]
Area of residence (ref: Urban)	1	1	1	1	1	1	1
rural	1.34 [0.61, 2.96]	1.50 [0.94, 2.36]	1.06 [0.43, 2.66]	1.39 [0.74, 2.61]	1.64 [0.40, 6.69]	1.20 [0.72, 1.98]	1.85 [0.59, 5.75]
Wealth index (ref: Lowest)	1	1	1	1	1	1	1
Second	1.03 [0.63, 1.69]	1.8 [0.92, 3.54]	1.67 [0.74, 3.8]	0.95 [0.46, 1.95]	1.72 [0.38, 7.78]	1.64 [0.78, 3.47]	1.61 [0.63, 4.09]
Third	**0.42 [0.23, 0.77]**^c^	0.64 [0.3, 1.35]	0.45 [0.13, 1.5]	**0.48 [0.3, 0.77]**^d^	1.75 [0.30, 10.18]	0.75 [0.45, 1.25]	**0.18 [0.03, 0.97]**^c^
Fourth	0.52 [0.26, 1.04]	1.22 [0.66, 2.26]	0.9 [0.36, 2.22]	0.89 [0.57, 1.38]	0.38 [0.06, 2.42]	1.4 [0.85, 2.31]	0.59 [0.27, 1.3]
Highest	**0.29 [0.14, 0.61]**^d^	**0.41 [0.25, 0.7]**^d^	**0.33 [0.12, 0.9]**^c^	0.59 [0.27, 1.29]	0.72 [0.08, 6.18]	0.85 [0.44, 1.65]	0.35 [0.1, 1.19]
Inflammation (ref: no inflammation)	1	1	1	1	1	1	1
Chronic or acute inflammation	**2.03 [1.03, 4.01]**^c^	**17.76 [7.78, 40.55]**^d^	**3.89 [1.59, 9.48]**^d^	1.63 [0.86, 3.07]	**7.86 [1.61, 38.30]**^c^	2.06 [0.62, 6.82]	**14.17 [6.37, 91.72]**^d^
**Growth indices**							
Non-stunted	1	1	1	1	1	1	1
Stunted (ref: non-stunted)	1.81 [0.93, 3.51]	1.21 [0.59, 2.49]	1.81 [0.82, 4.00]	1.51 [0.90, 2.53]	3.96 [0.97, 16.18]	**2.02 [1.30, 3.13]**	**3.44 [1.07, 11.09]**
sNormal weight	1	1	1	1	1	1	1
Overweight (ref: normal weight)	0.68 [0.40, 1.18]	1.16 [0.68, 2.00]	1.21 [0.51, 2.88]	**0.76 [0.59, 0.99]**^c^	2.04 [0.81, 5.12]	0.75 [0.46, 1.24]	0.86 [0.41, 1.82]
Thinness (ref: normal weight)	1.44 [0.74, 2.8]	0.48 [0.16, 1.43]	0.58 [0.13, 2.49]	1.04 [0.56, 1.95]	**9.15 [2.28, 36.68]**^d^	1.16 [0.56, 2.41]	0.99 [0.22, 4.37]

CI: confidence interval; COR: crude odds ratio; CRRR: crude relative risk ratio; ID: iron deficiency; IDA: iron deficiency anaemia; MND: micronutrient deficiencies.

a Anaemia was defined as haemoglobin <115 g/L and <120 g/L for 10–11 years old and 12–18 years old, respectively. ID was defined as serum ferritin<15 μg/L or 70 μg/L for apparently healthy individuals and individuals with inflammation [CRP > 5 mg/L and/or AGP > 1 mg/L], respectively; IDA was defined as the co-existence of anaemia and ID. Low serum zinc was defined as serum zinc under different conditions according to IZiNCG; Low serum retinol was defined as serum retinol concentration <0.70 μmol/L.

b Any MNDs referred to having any of the following micronutrient deficiencies: iron deficiency, low serum zinc or retinol.

c p < 0.05.

d p < 0.01.

**Table 4:** Adjusted binominal and multi-nominal logistic regressions between micronutrient status and growth indices among female adolescents aged between 10 and 18 years old (n = 1471).

	Individual MNDs^a^					Any MNDs^b^	
	Anaemia	ID	IDA	Low serum zinc	Low serum retinol	1 MND	≥2 MNDs
	AOR [95% CI]	AOR [95% CI]	AOR [95% CI]	AOR [95% CI]	AOR [95% CI]	ARRR [95% CI]	ARRR [95% CI]
Non-stunted	1	1	1	1	1	1	1
Stunted (ref: non-stunted)	1.42 [0.80, 2.51]	1.07 [0.54, 2.11]	1.40 [0.65, 3.01]	1.54 [0.91, 2.61]	**8.92 [2.26, 35.15]**^c^	**2.06 [1.31, 3.25]**^c^	2.30 [0.56, 9.51]
Normal weight	1	1	1	1	1	1	1
Overweight (ref: normal weight)	0.68 [0.36, 1.31]	1.06 [0.50, 2.25]	1.14 [0.47, 2.73]	0.79 [0.58, 1.06]	3.57 [0.78, 16.38]	0.73 [0.44, 1.23]	0.74 [0.32, 1.70]
Thinness (ref: normal weight)	1.71 [0.97, 2.99]	0.49 [0.19, 1.25]	0.61 [0.13, 2.83]	1.15 [0.58, 2.28]	**12.25 [3.47, 43.33]**^c^	1.24 [0.57, 2.68]	1.19 [0.31, 4.56]

All analyses were adjusted for confounders including age, ethnicity, area, wealth index, and inflammation.

AOR: adjusted odds ratio; ARRR: adjusted relative risk ratio; CI: confidence interval; ID: iron deficiency; IDA: iron deficiency anaemia; MND: micronutrient deficiencies.

a Anaemia was defined as haemoglobin <115 g/L and <120 g/L for 10–11 years old and 12–18 years old, respectively. ID was defined as serum ferritin<15 μg/L or 70 μg/L for apparently healthy individuals and individuals with inflammation [CRP > 5 mg/L and/or AGP >1 mg/L], respectively; IDA was defined as the co-existence of anaemia and ID. Low serum zinc was defined as serum zinc under different conditions according to IZiNCG; Low serum retinol were defined as serum retinol concentration <0.70 μmol/L.

b Any MNDs referred to having any of the following micronutrient deficiencies: iron deficiency, low serum zinc or low serum retinol.

c p < 0.01.

Our findings from bivariate logistic regression showed that while female adolescents aged 13–15 years old had increased odds of ID (COR = 2.24 [1.44, 3.47], p < 0.01), this risk was higher in those aged 16–18 (COR = 2.99 [1.70, 5.27], p < 0.01), and late-adolescents also had significantly higher risk of having more than one MND (CRRR = 3.15 [1.02, 9.80], p < 0.05; Table 3). Inflammation status was associated with increased odds of anaemia (COR = 2.03 [1.03, 4.01], p < 0.05), ID (COR = 17.76 [7.78, 40.55], p < 0.01), and low serum retinol (COR = 7.86 [1.61, 38.30], p < 0.05), but had no impact on odds of low serum zinc (Table 3). Ethnic minorities had increased odds of low serum zinc (COR = 1.63 [1.08, 2.47], p < 0.05); whereas female adolescents from the wealthiest quintile were significantly protected against IDA (COR = 0.33 [0.12, 0.9], p < 0.05). Surprisingly given the differences in prevalence (Supplementary Figs. S3 and S4), no significant association was found between MNDs and rural or urban areas of residence in this population (Table 3).

In the multivariate logistic regression models, after adjusting for age, ethnicity, area of residence, wealth index and inflammation; stunting and thinness were associated with increased odds of low serum retinol (stunting: AOR: 8.92 [2.26, 35.15], p < 0.01; thinness: AOR: 12.25 [3.47, 43.33], p < 0.01). In addition, stunting increased odds of having at least one MND (ARRR: 2.06 [1.31, 3.25], p < 0.01) compared to those without any MNDs (Table 4). However, it is important to note that the prevalence of low serum retinol among the study participants was extremely low (1.5%, n = 16; Table 4), thus these results should be interpreted with caution. We found no significant impact of any growth indices on the odds of low serum zinc (Table 4). In the unadjusted bivariate logistic regression, overweight appeared protective against low serum zinc (COR: 0.76 [0.59, 0.99], p = 0.04; Table 3). However, this association disappeared after adjusting for covariates (Table 4). Similarly, no significant association was found between overweight and any MNDs in the multivariate logistic regression models. The results of secondary analysis on the randomly generated subsamples (50%) were consistent with the results on the full dataset reported above.

## Discussion

This is the first study to investigate the prevalence and associations between MNDs and nutritional status (i.e., both under- and overnutrition), in contemporary female adolescents living in Vietnam. Our data starkly illustrate the current double burden of malnutrition in Vietnam, highlighting a high prevalence of overweight (27%) co-existing alongside undernutrition (17.3%; combined prevalence of either stunting, 14.3%, and/or thinness, 6.9%) among female adolescents. Low serum zinc was the most commonly observed MND, affecting 40% of participants in this cross-sectional cohort, followed by ID (13.4%). In contrast, low serum retinol was rarely observed (1.5%). Notably, stunting significantly increased the risk of having any MND (ARRR: 2.06 [1.31, 3.25]), and the prevalence of stunting still predominated over overweight (26.8% versus 22%) in late adolescent females aged 16–18 years old living in Vietnam in 2020.

Between 2010 and 2020 the prevalence of overweight in children and young people (5–19 years old) living in Vietnam was estimated to have more than doubled (8.5–19%).^7^ Strikingly, our study suggests an even higher prevalence of overweight among female adolescents living in Vietnam between 2019 and 2020, reaching 27%. This is higher than most LMICs in the South-Eastern Asia region,^7^ and likely stems from a marked nutrition transition in Vietnam that has come secondary to a period of rapid and sustained economic growth with large increases in household wealth.^7^^,^^17^^,^^18^ Our study illustrates the scale of the double burden of malnutrition in female adolescents in Vietnam, as a comparable percentage of participants in this nationally-representative survey were undernourished (stunting, thinness, MNDs) as were overweight. This emergent public health problem has been prioritised in the National Nutrition Strategy for 2021–2030.^17^

Significant demographic differences were observed between stunted and non-stunted adolescents, these included participant’s areas of residence (urban versus rural), geographical regions, ethnicity, and wealth index. These factors are often interrelated, as in Vietnam many ethnic minorities live in rural or mountainous areas, with limited education, household income and diet choice. Minority groups are much more likely to live in moderate or severe food insecurity, and have less access to health care services including vaccinations, as well as the improved sanitation and water supply found in urban regions.^17^^,^^19^ Ethnic minorities consequently have a higher prevalence of parasitic infections and diarrhoea, leading to increased risk of stunting in early childhood (under 5 years of age).^20^ Therefore, in the Vietnam Nutrition Strategy 2021–2030, ethnic minorities were seen as a critical target population to be individually evaluated for most nutrition targets.^17^ Our data showing that wealth index was higher in non-stunted adolescents aligns with previous research that linked household wealth to protection from childhood stunting in developing countries including Vietnam.^21^

Interestingly, we found no significant association between body weight status (thinness, normal, or overweight) and either wealth index, ethnicity, geographical regions, or areas of residence. That is, the results show a high prevalence of overweight among Vietnamese female adolescents irrespective of their sociodemographic factors. Although social inequalities are linked to obesity, the associations between overweight and socioeconomic status are complex, gender- and population-dependent (e.g., higher risk of obesity has been associated with social inequalities in high income countries, but associated with wealth in LMIC), and have been shown to shift overtime with economic development.^22^ To date there are very limited data from female adolescents in Vietnam to draw comparisons to. For example, a longitudinal study of Vietnamese children (mean age 8 years at baseline and 15 years at follow-up) found children in the top tertile of household wealth had a much higher relative risk of overweight and obesity in comparison to those in the bottom tertile.^23^ However, this mixed cohort (n = 999; 50.2% males) within a larger multi-country study of poverty and inequality, the Young Lives study, had very low cumulative incidence of overweight (0.5–2.5%) overall.^23^ Moreover, in contrast to the GNS 2020, it was not designed as a nationally representative survey. Rather, the Young Lives study deliberately used a semi-purposive sampling strategy with an over-sampling of poor communes that resulted in an underrepresentation of urban areas in relation to both total population share and level of development, likely explaining the discordance in findings.

In addition to negatively impacting immune function and increasing the risk of morbidity for the adolescents themselves, MNDs, in female adolescents in particular, can lead to intergenerational consequences through shaping foetal programming, development in early life, and the cardiometabolic health of the offspring in the long term.^3^ Our data highlights a high prevalence of low serum zinc levels and ID in Vietnamese female adolescents, with those from higher household wealth being protected from ID, IDA, and all-cause anaemia. In addition, female adolescents from minority ethnic groups were more likely to have low serum zinc, likely related to both increased prevalence of food insecurity, infections, and diarrhoea, as well as consumption of more staple and starchy foods that are lower in essential micronutrients.^19^ Our study highlights that tailored strategies are necessary to address disparities in nutritional status in adolescent females from minority ethnic groups and households with lower wealth indices in Vietnam.

In contrast to the persistently high prevalence of low serum zinc levels and ID, there was a much lower prevalence of low serum retinol among the female adolescent participants in the GNS2020. Previous work examining the 2016 Vietnam Household Living Standards Survey suggests eggs are the primary source of own-produced vitamin A and fat in Vietnamese households,^24^ and nationally total egg production was four times higher in 2020 in comparison to 2000, which might explain these results.^25^ In addition, in 2016 the government signed a decree (09/2016/ND-CP) mandating fortification of vegetable oils, consumed by 90% of WRA in Vietnam daily, with Vitamin A. This decree also called for salt to be fortified with iodine and the addition of iron and zinc to wheat flour. However, as noted by the WHO and UNICEF in 2021, this has not been fully implemented perhaps explaining the persistence of low serum zinc and ID in this study.^26^

Notably, we found that MNDs were generally more prevalent among older adolescents (16–18 years old), which may reflect increased demands for both micro- and macronutrients during growth spurts and sex maturation during puberty. In addition, repeated blood loss during menstruation will deplete iron stores particularly for females with limited access to iron-rich foods, or who have low iron absorption, which leads to ID with or without anaemia.^27^ Strikingly, our data show that the prevalence of iron deficiency was four times higher (32% versus 8%) among older female adolescents from rural compared to urban areas, whereas the differences in prevalence between rural and urban in the two younger age groups were not as marked (9.3% versus 7.8% for 10–12 year olds, and 19.2% versus 14.6% for 13–15 year olds). Early marriage (under 18 years of age) in the north midland and mountainous and central highland regions remains common (21.1% and 18.1% respectively) and approximately twice the national average.^17^ Therefore, early pregnancy and childbirth may explain the higher prevalence of ID in older rural female adolescents. Beyond the fact that, even in the absence of frank anaemia, ID will be symptomatic for many, these data are a public health concern as iron sufficiency during pregnancy is essential for both maternal and foetal health, and increases risk of low birth rate and poor pregnancy outcomes.^28^ Although with economic growth, the purchasing capacity and dietary pattern of Vietnamese has changed to include more meat, vegetables and less starchy food at a national level, stark differences in household expenditure on food groups exist between ethnic minority, rural, and poorer households,^6^^,^^19^ which likely also contribute to the disparities in MND between rural and urban female adolescents observed here.

In this study, we did not find any association between overweight and MNDs, although overweight, in particular obesity, has been shown to increase risk for ID in children and young people in meta-analysis OR = 1.51 (1.20, 1.82).^29^ The possible explanation could be the extremely low prevalence of obesity (2.7% in urban and 1.3% in rural area) in our population in comparison to those included in the meta-analysis, which were from North America and European populations. Our results match those of Laillou and colleagues,^30^ who concluded overweight/obesity was not a risk factor for ID in a large cohort of Vietnamese women (n = 1526).

As with all observational studies, our cross-sectional study has limitations, perhaps most notably that it cannot address causality. Statistically, type 2 errors are always a risk where numerous analyses are performed and where variables are likely not completely independent of each other (e.g., stunting, thinness and MNDs). In addition, not all age and sex groups were equally recruited, blood samples were limited in volume in some cases, and serum retinol measurement was performed only on a subset of GNS participants. We note that as women’s health was a key component of the GNS2020 study design, far fewer adolescent male participants were recruited (n = 229), which precluded them as a potential comparator group. Similarly, fewer participants recruited to the GNS 2020 were in late adolescence, and these age and sex groups should be a particular focus for data collection in the next GNS survey.

Not least, an important limitation in examining this age group was that due to missing values we could not include puberty stage as a confounder in regression analyses. Nonetheless, our study had notable strengths. The GNS 2020 was a nationally-representative population-based survey and findings can be generalised to female adolescents of the same age group across Vietnam. Moreover, the measurement of biochemical biomarkers of both micronutrient status and inflammation permitted following international best practice guidelines for defining MNDs, and examining these data alongside anthropometric and socio-demographic variables provides unprecedented insights into the current double burden of malnutrition among female adolescents in Vietnam.

Robust, timely and reliable data on the burden of MNDs are essential to inform, design and implement successful nutrition policies and programmes aimed at reducing malnutrition. Our study has implications for addressing social determinants of MNDs and provides key data for supporting nutritional policy and practice in regard to prevention, early identification of malnutrition, as well as addressing socioeconomic inequities. In addition to enforcing existing food fortification policies to reduce MNDs,^26^ food environment and food systems approaches should be considered to stem the recent stark increase in overweight in young people living in Vietnam and other Western Pacific nations newly facing the rapid increase of overnutrition, such as the Philippines and Cambodia.

In conclusion, our data highlight that more female adolescents were living with overnutrition than undernutrition in Vietnam in 2020. However, undernutrition and low serum zinc remain persistently prevalent. Regional, residential, ethnic, and wealth disparities in undernutrition and MND suggest the needs for targeted interventions to ensure equitable access for all and to meet local needs. These data should aid in prioritizing public health and nutrition education initiatives, and provides reference points for assessing the effectiveness of future interventions.

## Contributors

XT, PYT, SVS, YYG, and JBM designed and conceptualized the study. YYG and JBM supervised the study. NTT and VKT were responsible for GNS 2020 data curation, investigation, and methodology. SDN and DTT performed data cleaning and final statistical analyses. NTT, VKT, SDN and DTT had full access to all the data and took responsibility for the integrity of the data. XT, PYT and SVS wrote code for formal analyses and performed statistical analyses on data subsets. XT prepared the tables and figures and original manuscript draft. JBM critically revised the manuscript and all authors reviewed the manuscript and approved the submitted final version.

## Data sharing statement

The GNS 2020 dataset is the property of the National Institute of Nutrition, Vietnam, and is not public available. This secondary analysis was conducted under a partnership agreement between the University of Leeds and National Institute of Nutrition of Vietnam. Requests to access the datasets should be directed to ninvietnam@viendinhduong.vn.

## Declaration of interests

We declare that we have no conflicts of interest.

## Appendix ASupplementary data

Supplementary Figs. S1–S4 and Table S1

Tan_Abstract_Vietnamese
