# Supplementary material for: Micronutrient deficiencies and the double burden of malnutrition in Vietnamese female adolescents: a national cross-sectional study in 2020
Source: Lancet Reg Health West Pac. 2024 Aug 7;50:101164. doi: 10.1016/j.lanwpc.2024.101164 (PMC11363818; doi:10.1016/j.lanwpc.2024.101164)
Supplement: Supplementary Figs. S1–S4 and Table S1 [file mmc1.pdf]

SUPPLEMENTARY MATERIAL

Micronutrient deficiencies and the double burden of malnutrition in Vietnamese female adolescents: a national cross-sectional study in 2020

Authors

X. Tan, P.Y. Tan, S.V. Som, S.D. Nguyen, D.T. Tran, N.T. Tran, V.K. Tran, J. B. Moore* and Y. Y. Gong

*Corresponding Author: J.B.Moore@leeds.ac.uk

Table of Contents

Figure S1 Participant Flow Diagram 2

Table S1 Number and percentage missed observations in dataset 3

Figure S2 Prevalence of overweight, stunting and thinness in rural and urban areas 4

Figure S3 Prevalence of micronutrient deficiencies in rural and urban areas 5

Figure S4 Prevalence of any MNDs in rural and urban areas 6

FIGURE S1

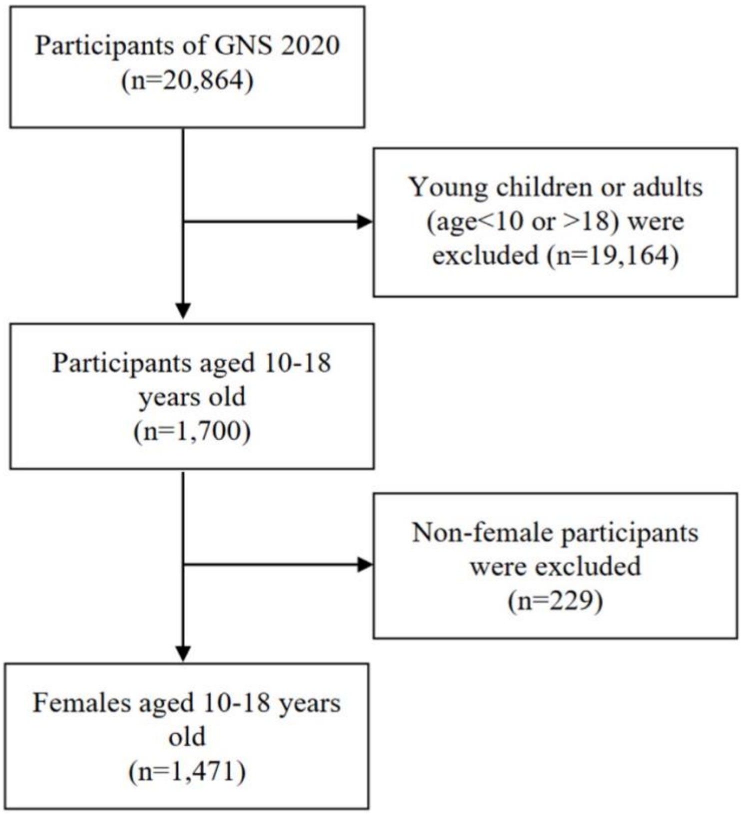

Figure S1 Participant Flow Diagram

Table S1 Number and percentage missed observations in dataset

	Variable	Number of observations	Number of missed observations	Percentage of missed observations
**Age**	age in year	1471	0	0.0
**Anthropometry**	body weight	1471	0	0.0
	body height	1253	218	14.8
**MNDs & inflammation**	anaemia	1396	75	5.1
	ID	1296	175	11.9
	IDA	1396	75	5.1
	low serum zinc	1389	82	5.6
	low serum retinol	1133	338	23.0
	number of MNDs	868	603	41.0
	inflammation status	1297	174	11.8
**Socioeconomic**	ecological region	1471	0	0.0
	residence of living	1471	0	0.0
	ethnicity	1471	0	0.0
	wealth index	1471	0	0.0
**Others**	puberty	212	1259	85.6

ID: iron deficiency; IDA: iron deficiency anaemia; MNDs: micronutrient deficiencies.

FIGURE S2

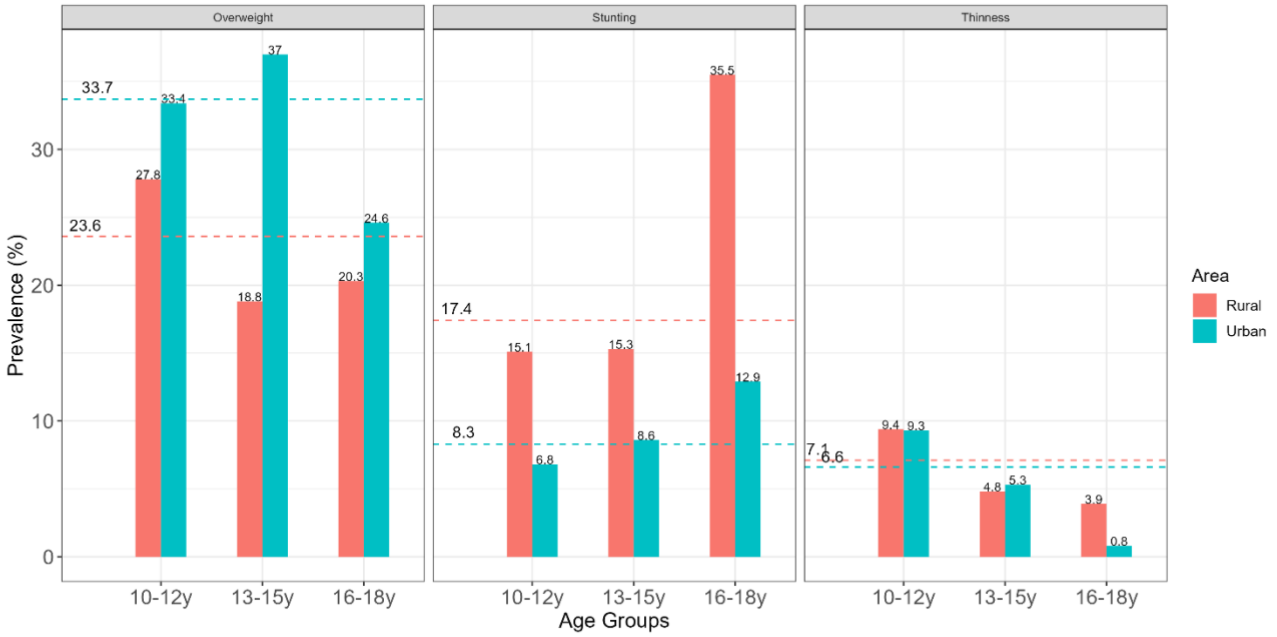

Figure S2 Prevalence of overweight, stunting and thinness in rural and urban areas. Overweight: BMI-for-age z score (BAZ) > +1 SD; stunting: height-for-age z score (HAZ)< -1 SD; thinness: BAZ< -2 SD. Prevalence was estimated based on sampling weights. Bars represent the prevalence in each age groups. Dashed lines illustrate the overall prevalence.

FIGURE S3

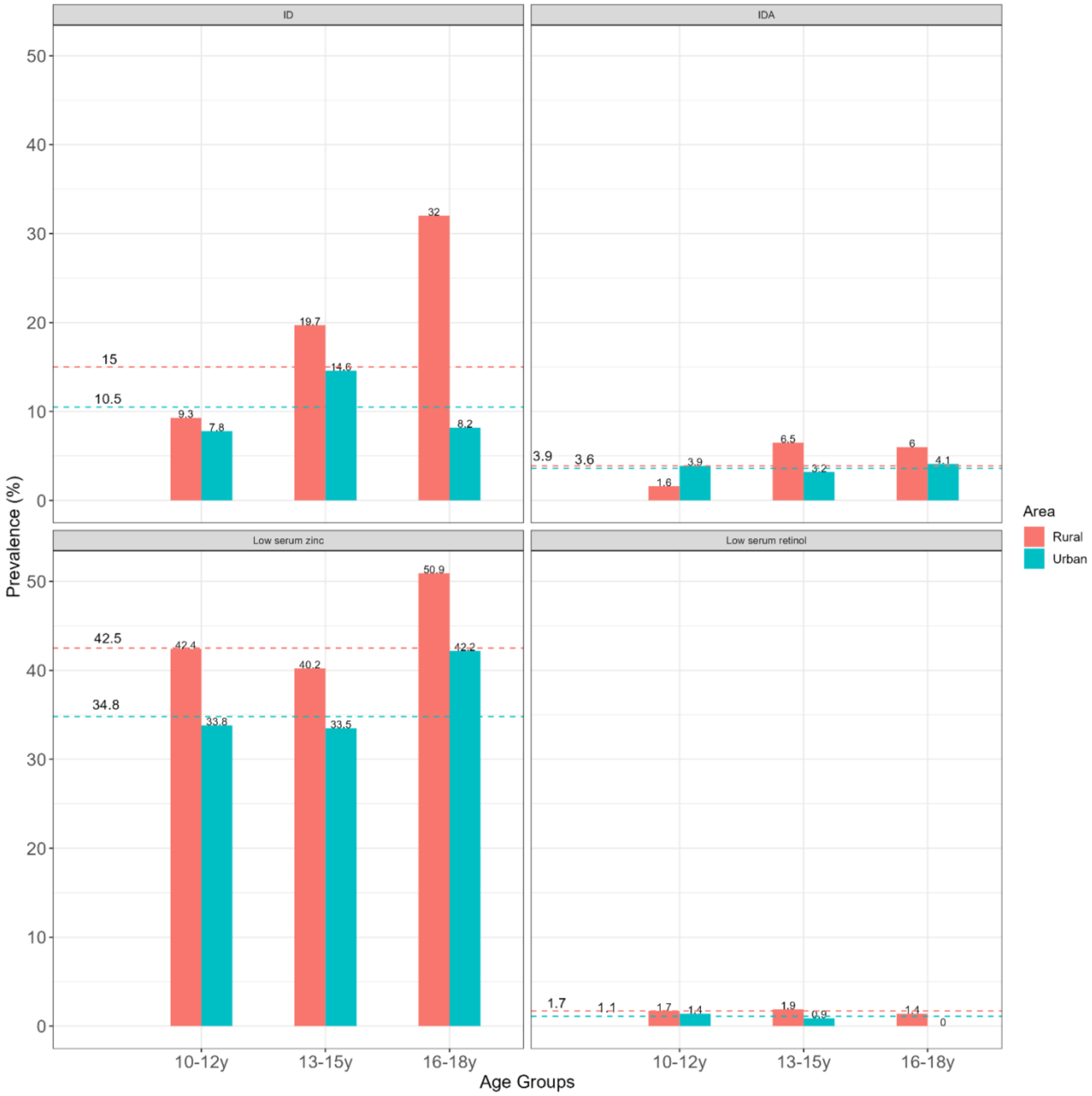

Figure S3 Prevalence of micronutrient deficiencies in rural and urban areas. ID: iron deficiency, IDA: iron deficiency anaemia.

FIGURE S4

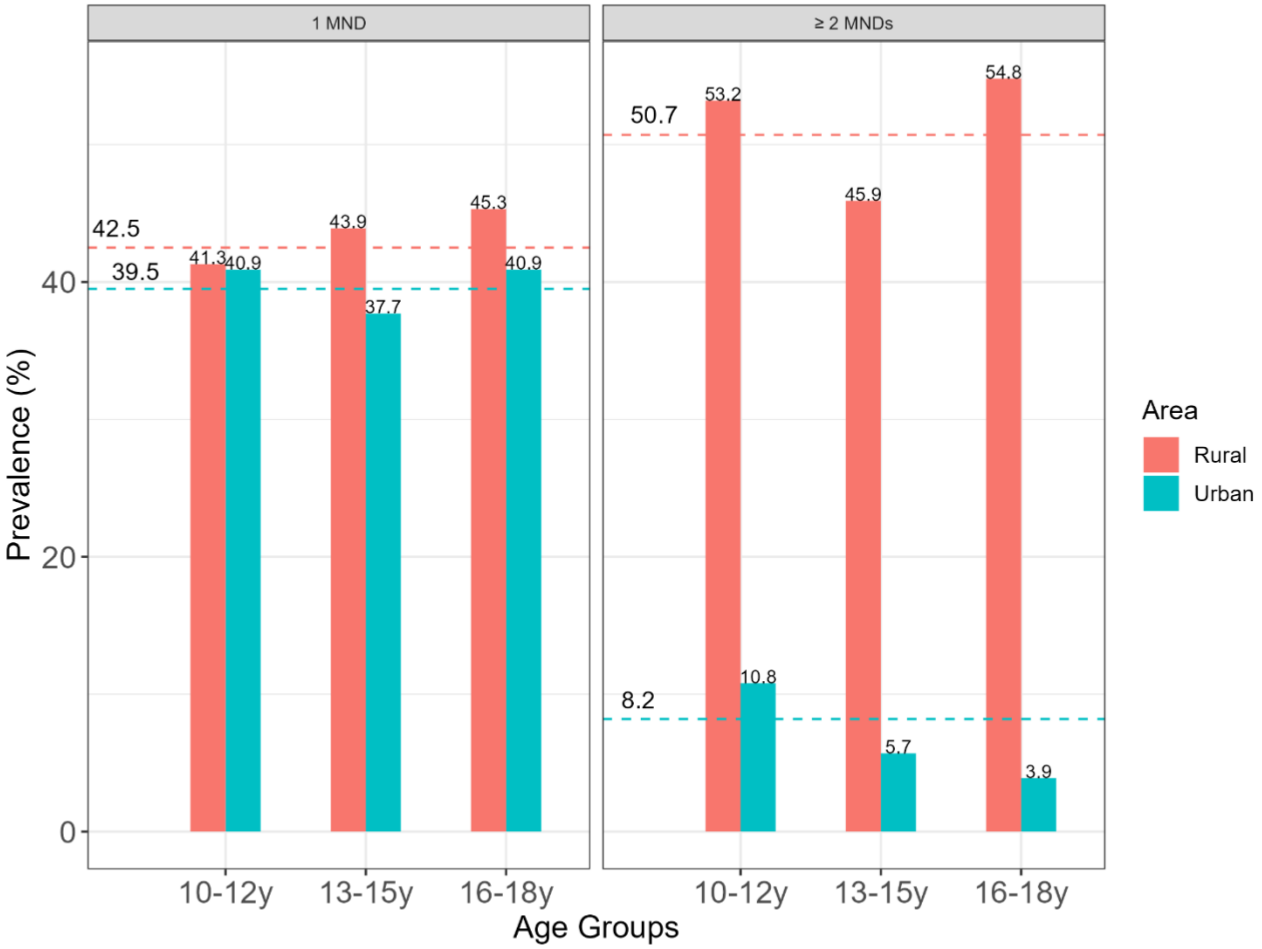

Figure S4 Prevalence of any MNDs in rural and urban areas. MNDs included any of: iron deficiency, low serum zinc and low serum retinol. MNDs: micronutrient deficiencies.
