# Supplementary material for: Micronutrient deficiencies and the double burden of malnutrition in Vietnamese female adolescents: a national cross-sectional study in 2020
Source: Lancet Reg Health West Pac. 2024 Aug 7;50:101164. doi: 10.1016/j.lanwpc.2024.101164 (PMC11363818; doi:10.1016/j.lanwpc.2024.101164)
Supplement: Tan_Abstract_Vietnamese [file mmc2.pdf]

TIÊU ĐỀ

Thiếu vi chất dinh dưỡng và gánh nặng kép về suy dinh dưỡng ở nữ vị thành niên Việt Nam: một nghiên cứu cắt ngang quốc gia năm 2020

TÁC GIẢ

X. Tan, P.Y. Tan, S.V. Som, S.D. Nguyen, D.T. Tran, N.T. Tran, V.K. Tran, J. B. Moore* and Y. Y. Gong

*Địa chỉ tương ứng: J.B.Moore@leeds.ac.uk

TÓM TẮT

Đặt vấn đề: Việt Nam đang phải đối mặt với gánh nặng kép về suy dinh dưỡng, với tỷ lệ thừa cân ngày càng tăng cùng với tình trạng suy dinh dưỡng (thấp còi và/hoặc gầy còm) và thiếu vi chất dinh dưỡng (MND). Mặc dù tình trạng suy dinh dưỡng ở nữ giới ở tuổi vị thành niên dẫn đến tình trạng sức khỏe kém cùng với những tác động tiềm ẩn ảnh hưởng đến thế hệ sau, nhưng vẫn chưa có một nghiên cứu toàn diện nào điều tra về thiếu vi chất dinh dưỡng và tình trạng dinh dưỡng ở nữ thanh thiếu niên Việt Nam hiện nay.

Phương pháp: Số liệu của nữ vị thành niên từ 10 đến 18 tuổi (n=1,471) tham gia vào Tổng điều tra dinh dưỡng quốc gia Việt Nam năm 2020 đã được phân tích. Các chỉ số sinh học dinh dưỡng trong máu, số đo nhân trắc học và dữ liệu nhân khẩu xã hội được thu thập, và mối liên quan giữa tình trạng dinh dưỡng và MND được phân tích; trong đó thiếu máu, thiếu sắt, thiếu máu do thiếu sắt, nồng độ kẽm huyết thanh thấp, nồng độ retinol huyết thanh thấp và bất kỳ tình trạng MND nào được xem là kết quả đầu ra cụ thể.

Kết quả: Tỷ lệ thừa cân, thấp còi, và gầy còm lần lượt là 27,2%, 14,3% và 6,9%. Tỷ lệ nữ vị thành niên có nồng độ kẽm huyết thanh thấp là phổ biến với 39,8%, cũng như thiếu sắt là 13,4%. Các phân tích hai biến cho thấy tuổi lớn hơn (16-18 tuổi), dân tộc thiểu số, chỉ số giàu nghèo thấp hơn và tình trạng viêm có liên quan đến MND. Trong mô hình hồi quy logistic đã điều chỉnh, tình trạng thấp còi có liên quan với tăng 8,92 lần tỷ lệ có retinol huyết thanh thấp với khoảng tin cậy 95% [2,26, 35,15], p<0·01, cũng như tăng 12,25 lần tỷ lệ gầy còm với khoảng tin cậy 95% [3,47, 43,33], p<0·01. Suy dinh dưỡng thể thấp còi cũng liên quan đến việc tăng tỷ lệ MND (2,06 [1·31, 3·25], p<0·01).

Bàn luận: Ở Việt nam, nhiều nữ vị thành niên bị thừa cân hơn so với thiếu dinh dưỡngvào năm 2020. Tuy nhiên, tình trạng suy dinh dưỡng, tỷ lệ nữ có nồng độ kẽm huyết thanh thấp và thiếu sắt vẫn còn phổ biến. Các phương pháp tiếp cận hệ thống thực phẩm cần được cân nhắc nhằm ngăn chặn sự gia tăng rõ rệt gánh nặng kép về suy dinh dưỡng ở giới trẻ Việt Nam.

Nguồn tài trợ: BBSRC BB/T008989/1 của Vương quốc Anh.
